# Letter in Reference to the Use of Sulphuric Ether, &c.

**Published:** 1847-06

**Authors:** G. W. Clippinger

**Affiliations:** Terre Haute, Indiana


ARTICLE VIII.

*Letter in Reference to the use of Sulphuric Ether*, &c. From G. W. Clippinger, M. D., of Terre Haute, Indiana.

Professor Evans:

*Dear Sir:*—Soon after my return, an opportunity presented for an experiment with the sulph. ether. Having failed to procure the rectified, I used the ordinary article of the shops; supposing the only difference in the two articles would be, in the latter requiring a longer period for its effects to be experienced. In this particular there was no disappointment. After inhaling the ether some five minutes, a sensation of numbness was complained of, which, in the course of a few minutes more, was succeeded by a state of complete insensibility. This condition continued five minutes, during which time no perceptible change in the pulse was detected; the respiration was easy and natural, the subject exhibiting the appearance of a person in a profound sleep. No evidence of pain from the prick of a lancet or pinching could be noticed. The arm, when elevated, would drop the instant let go; the mouth opened, would remain so until closed; the pupil of the eye remained unaltered—was neither dilated nor contracted. At the expiration of five minutes, this state of insensibility was succeeded by that of delirious excitement. The subject of experiment having combativeness well developed, you can readily guess he kept me employed for some minutes—perhaps fifteen—during which time he was thrown upon the bed, and held until the effects entirely passed off, leaving a sense of depression with some headache. I have exhibited the same preparation of sulph. ether in two cases since; in both the effects were similar to those described, excepting the length of time the subsequent excitement lasted — which, in one of the last two experiments continued an hour and a half. Dr. Hitchcock was present during one of the experiments, and although desirous of obtaining the first effect of the ether in an operation he wishes to perform, he declines doing so on account of the fears entertained of the latter. In your experiments, has the subsequent excitement been present to an unpleasant degree? Or have you ever used the ordinary sulph. ether of the shops in an experiment? If a convenient opportunity presents, please send me some of the preparation of ether used by yourself; as I am desirous to test its effects to my entire satisfaction.

*A Somewhat Novel Case* came under my observation some two weeks since. The patient was a widower, aged about forty. He addressed me as I was walking by, and said he believed he had made an eunuch of himself. He was requested to return home, where I shortly after saw him, and to my surprise, after undoing various old cloths from about the scrotum, one testicle hung suspended by the cord on its anterior and inferior surface, entirely exposed, covered with dirt, considerably tumefied, and very tender. After removing as much of the dirt as was practicable, with much difficulty the testicle was returned, and the wound closed by aid of sutures and adhesive plaster. Notwithstanding the testicle had been thus exposed two hours, suspended by the cord, covered by dirt, somewhat tumefied, and the patient having walked a mile with it in that condition, the wound was entirely cicatrized in three days, and the patient resumed his employment. The patient could give no satisfactory account of how the wound had been received, and only said it was done with a razor.

*May 5th*, 1847.

[I have given the ether in a great number of cases, but have, fortunately, not met with a single instance in which the excitement was manifested during the subsidence of its narcotic effects. Several cases of the kind have been reported, but they are quite few in comparison to the great number in which no such effects have followed. I have used the rectified generally; but once got an article that was so diluted as to be of no use.

The ether should be rectified, to remove impurities that might have a deleterious influence; but I know of no impurity that would cause it to have the effect above referred to. E.]

